# Hydrothermal Synthesis of Siderite and Application as Catalyst in the Electro-Fenton Oxidation of *p*-Benzoquinone

**DOI:** 10.3390/molecules27228056

**Published:** 2022-11-20

**Authors:** Özkan Görmez, Barış Saçlı, Uğur Çağlayan, Dimitrios Kalderis, Belgin Gözmen

**Affiliations:** 1 Department of Chemistry, Arts and Science Faculty, Mersin University, 33343 Mersin, Turkey; 2 Central Research Laboratory of Çukurova University (CUMERLAB), Çukurova University, 01330 Adana, Turkey; 3 Department of Electronic Engineering, Hellenic Mediterranean University, 73133 Chania, Greece

**Keywords:** electro-Fenton, siderite, ferrous carbonate, benzoquinone, wastewater treatment

## Abstract

A weak aspect of the electro-Fenton (EF) oxidation of contaminants is the dependence of the Fenton reaction on acidic pH values. Therefore, the rationale of this work was to develop a novel catalyst capable of promoting the EF oxidation process at near-neutral and basic pH values. In this framework, rhombohedral FeCO_3_ was synthesized hydrothermally and used as a catalyst in the EF oxidation of *p*-benzoquinone (BQ). The catalyst was characterized using various surface and spectroscopic methods. Moreover, the effects of applied current (100–500 mA), time (1–9 h), catalyst dosage (0.25–1.00 g L^−1^), and initial concentration of BQ (0.50–1.00 mM) on the total organic carbon removal efficiency were determined. The results indicated that a 400 mA current was sufficient for a 95% total organic carbon removal and that the increase in catalyst dosage had a positive effect on the mineralization of BQ. It was determined that at pH 3, FeCO_3_ behaved like a homogeneous catalyst by releasing Fe^3+^ ions; whereas, at the pH range of 5–7, it shifted to a homogeneous/heterogeneous catalyst. At pH 9, it worked solely as a heterogeneous catalyst due to the decrease of Fe ions passing into the solution. Finally, the spent catalyst did not undergo structural deformations after the EF treatment at higher pH values and could be regenerated and used several times

## 1. Introduction

*p*-Benzoquinone (IUPAC name cyclohexa-2,5-diene-1,4-dione, referred to as BQ thereof) is a naturally occurring, crystalline organic compound with a strong odor. Although BQ is a natural organic compound, it is also produced synthetically as it has several industrial applications. BQ is used directly in the dye, textile, pesticide, and drug industries and has been detected as residue in various industrial effluents [1]. As an example, BQ is one of the most frequently encountered compounds identified in the wastewater of the production of polyaniline (PANI) conductive polymer [2], and lignocellulose biorefinery processes [3]. Moreover, BQ is also one of the main intermediate products formed during the oxidation of various benzene derivatives [4].

Exposure to BQ may result in acute and chronic effects, such as eye irritation, conjunctiva, cornea discoloration, and acute inhalation toxicity [5,6]. Additionally, long exposure to *p*-Benzoquinone may cause serious genetic damage in humans, and its toxicity has been reported as 267 times that of phenol [1]. For these reasons, BQ has been added to the TRI-Listed Chemicals of the United States Environmental Protection Agency [7]. The LC_50_ value of BQ has been reported as <0.01 mg L^−1^ for *Escherichia coli* [4]. Moreover, according to the LC_50_ value of 0.125 mg L^−1^ for fish (96 h exposure), it is classified as an ecotoxin.

Its high toxicity and environmental recalcitrance render biological treatment processes inadequate and disrupt the functioning of biological wastewater plants [1]. Therefore, the efficient elimination of BQ and its derivatives requires the design and use of more effective treatment processes. The efficiency of advanced oxidation processes (AOPs) for wastewater treatment and the strong oxidation potential of the hydroxyl radicals (*E*^0^(^•^OH/H_2_O) = 2.8 V/SHE) for persistent contaminants have been well-established. Conventional AOPs are Fenton-based, O_3_-based, and photocatalyst-based processes. In the Fenton process, ferrous ions react with H_2_O_2_ to produce hydroxyl radicals, as follows [8]:

Fe^2+^ + H_2_O_2_ → Fe^3+^ + ^•^OH + OH^−^   *k* = 63 M^−1^ s^−1^ (1)

However, as seen in Equations (2) and (3), Fenton reagents can scavenge the generated hydroxyl radicals. Therefore, determining the optimal conditions is the most critical step [9]

Fe^2+^ + ^•^OH → Fe^3+^ + OH^−^ (2)

H_2_O_2_ + ^•^OH → HO_2_^•^ + H_2_O (3)

Based on the typical Fenton reactions, there are also three distinct types of applications: Fenton-like, photo-Fenton, and electro-Fenton [10,11]. In the electro-Fenton (EF) process, H_2_O_2_ is continuously electrogenerated due to reducing O_2_ at the suitable cathode in an acidic medium, as shown in Equation (4). In addition, the catalytic amount of Fe^2+^ added to the solution is oxidized to Fe^3+^ as a result of the Fenton reaction. However, it is reduced to Fe^2+^ at the cathode (Equation (5)) and participates in the Fenton reaction again [12]

O_2_ +2H^+^ +2e^−^ → H_2_O_2_ (*E*^0^ = 0.695 V/SHE) (4)

Fe^3+^ + e^−^ → Fe^2+^   (*E*^0^ = 0.770 V/SHE) (5)

The optimum working pH value for the electro-Fenton method has been determined as 2.8. The presence of iron ions in the form of hydroxides above this pH value weakens the oxidation power of this method and also leads to iron sludge precipitation [8,13]. Recently, heterogeneous electro-Fenton studies have attracted attention. The use of heterogeneous iron-containing catalysts, either alone or supported on modified carbon electrodes, has provided the opportunity to work in broader pH ranges. Some of these catalysts have been used as both pH regulators and Fe^2+^ ion sources. For the heterogeneous EF method, iron oxide [13,14], FeFe/CuFe layered double hydroxides [15,16], zero-valent iron [17,18], and iron-containing natural structures [19] have been investigated. For example, Fe_3_O_4_ containing Fe^2+^ and Fe^3+^ in its structure, as a stable heterogeneous catalyst, is effective and reusable in the EF process. The efficiency of an Fe_3_O_4_ heterogeneous catalyst, as free or loaded on graphene oxide, has been most effective at pH 3.0. However, it also acted as an effective catalyst at other pH values to degrade organic compounds, such as amoxicillin [20], aniline [21], chloramphenicol, and metronidazole [22]. In particular, natural pyrite (FeS_2_) effectively catalyzed the degradation of a synthetic azo dye [23], levofloxacin [24], tyrosol [25], and sulfamethazine [26] by adjusting the pH of the solution to suitable values for the EF process. Heidari et al. (2021) compared the efficiencies of naturally occurring minerals chromite (FeCr_2_O_4_), chalcopyrite (CuFeS_2_), and ilmenite (FeTiO_3_) as catalysts in the oxidation of the antibiotic cefazolin by the EF process, and it was determined that the chalcopyrite structure was more effective than the others [27]. The use of pyrite and chalcopyrite acted as buffers and regulated the pH of the medium in the range of 3–4 depending on the catalyst dosage, according to the Equations (6)–(9) [23,25,26]

2FeS_2_ + 7O_2_ + 2H_2_O → 2Fe^2+^ + 4SO_4_^2−^ + 4H^+^ (6)

2FeS_2_ + 14H_2_O_2_ → 2Fe^3+^ + 14H_2_O + 4SO_4_^2−^ + 2H^+^ (7)

FeS_2_ + 14Fe^3+^ + 8H_2_O → 15Fe^2+^ + 2SO_4_^2−^ + 16H^+^ (8)

CuFeS_2_ + 16Fe^3+^ + 8H_2_O → Cu^2+^ + 17Fe^2+^ + 2SO_4_^2−^ + 16H^+^ (9)

Moreover, Hajiahmadi et al. (2021) investigated the catalytic ability of five natural iron minerals, including hematite (Fe_2_O_3_), magnetite (Fe_3_O_4_), siderite (FeCO_3_), limonite (FeOOH·nH_2_O), and pyrite (FeS_2_), as natural sources of iron in the heterogeneous electro-Fenton process for the degradation of gemcitabine [28]. The authors obtained their optimum results with a pyrite catalyst. Hadjltaief et al. (2018) investigated the degradation of 4-chlorophenol (4-CP) by the photo-Fenton process using natural hematite and siderite as heterogeneous catalysts and reported complete mineralization of 20 mg L^−1^ 4-CP at pH 3 with siderite catalyst after 120 min [29]. Acisli et al. (2017) studied the effects of different milling times of the siderite mineral on the catalytic degradation of Reactive Yellow 81 dye by the ultrasound-assisted Fenton process and observed that its efficiency increased after 6 h of milling due to the reduced particle sizes [30]. At the same time, Yan et al. (2013) determined that trichloroethylene degradation efficiency was higher due to the simultaneous activation of hydrogen peroxide and persulfate by siderite compared to that obtained by hydrogen peroxide activation alone [31]. It has been suggested that the use of iron-containing minerals as catalysts may add several advantages to the Fenton process. To name a few, the catalyst can be removed by precipitation and filtration, it is reusable, the initial pH value of the wastewater can be expanded to a wider range (pH 5–9), and the process remains largely unaffected by the presence of inorganic carbonate [32]. Ganiyu et al. (2018) reported the pH values of several real wastewater pH values and stated that, in most cases, the pH value was neutral or slightly basic [33]. Therefore, provided that heterogeneous EF applications remove the pH limitation of the typical EF process, the applicability of this process will be improved without the need for pH adjustment.

It is known that the combination of Fe^2+^ and carbonate ions performed remarkably well in activating H_2_O_2_ under neutral and alkaline conditions [34,35]. Moreover, FeCO_3_ complexes have higher quantum yields and molar absorption coefficients at UV_254_ [36]. As mentioned earlier, the efficiency of natural siderite in electro-Fenton and photo-Fenton applications is promising, even more so following the reduction of the particle size by grinding.

In the laboratory, the synthesis of nano-sized FeCO_3_ is achieved through a surfactant-assisted hydrothermal reaction of iron sulfate and urea or iron sulfate and a carbonate source [37,38]. However, naturally occurring and laboratory-prepared siderite have different morphologies, surface area, crystallinity, and other physicochemical properties [39]. Therefore, it cannot be assumed that the latter will exhibit the same catalytic performance as the naturally occurring mineral and further research is required.

Given the proven applicability of natural siderite in EF applications, the rationale of this work was to investigate the potential of laboratory-prepared siderite to act as a catalyst in the electro-Fenton oxidation of a recalcitrant contaminant at a wide pH range. Therefore, rhombohedral shape FeCO_3_ was prepared by a facile hydrothermal method. The physicochemical and surface properties of the as-synthesized catalyst were investigated by different techniques such as X-ray diffraction (XRD), N_2_ adsorption/desorption analysis, Fourier-transform Infrared, and Raman spectroscopy, scanning electron microscopy combined with energy dispersive X-ray analysis (SEM-EDX). The catalytic efficiency of FeCO_3_ in the EF process was examined at the degradation of BQ at a near-neutral pH value. Furthermore, the mineralization of the BQ solution was monitored at different applied currents, pH values, times, and FeCO_3_ dosages. Finally, the pH regulation ability and reusability of FeCO_3_ and the amount of Fe ions leaching into the solution during the EF process were studied.

## 2. Results and Discussion

### 2.1. Characterization of FeCO_3_ Catalyst

**Figure 1:**
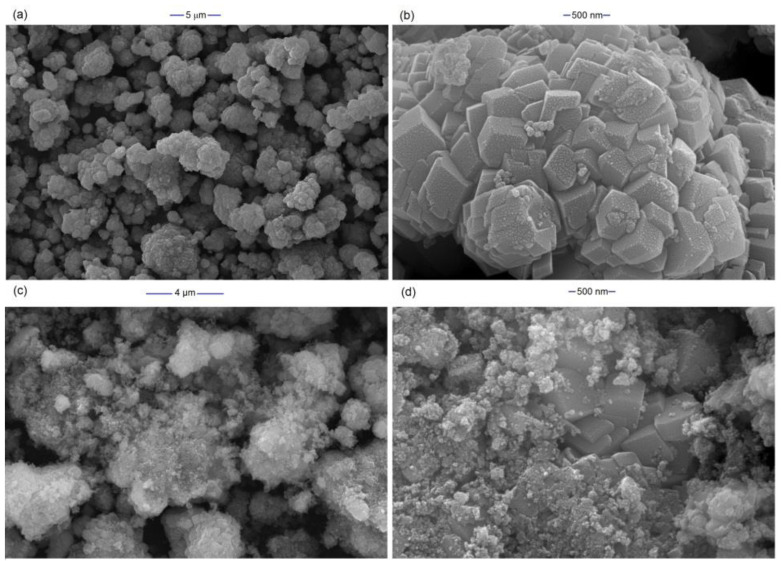
SEM images of as-prepared (**a**,**b**) and used (**c**,**d**) FeCO_3._ Operating conditions: pH = 5.6, 400 mA.

The SEM images of FeCO_3_, synthesized by the hydrothermal method at 140 °C, were depicted in Figure 1a,b. The SEM images showed agglomerates, composed of rhombohedral-shaped FeCO_3_ crystals in the size range of 300–500 nm. This observation comes in agreement with earlier works where laboratory-prepared siderite was applied in environmental remediation [40,41]. As shown in Figure 1c,d, the rhombohedral shape of the crystals was largely maintained after use, although covered with amorphous clusters of molecules of BQ and degradation products.

**Figure 2:**
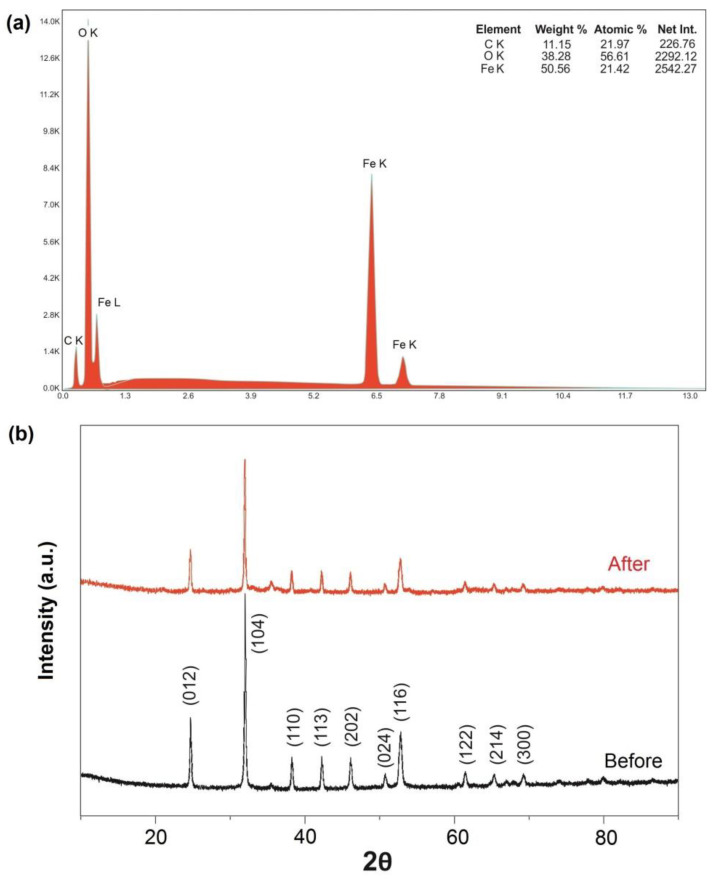
(**a**) EDS spectrum of FeCO_3_ and (**b**) XRD pattern of FeCO_3_ catalyst before and after the electro-Fenton process. Operating conditions: pH = 5.6, 400 mA.

EDS analysis results showed that the FeCO_3_ crystals were composed of Fe, C, and O atoms in a ratio close to 1:1:3 and did not contain any impurities (Figure 2a). In the natural siderite structure, Mn, Al, Si, and Mg often occur as impurities [29,30]. The XRD pattern of FeCO_3_, which included characteristic diffraction peaks of siderite, is presented in Figure 2b. The XRD patterns showed that the diffraction peaks at 2θ values of 24.7°, 32.0°, 38.2°, 42.3°, 46.1°, 50.7°, 52.6°, 61.4°, 65.3°, and 69.2° corresponded to (012), (104), (110), (113), (202), (024), (116), (122), (214), and (300) planes of the FeCO_3_, respectively. The pattern matches the typical crystal faces of siderite (JCPDS No. 00-029-0696) and recently reported patterns of laboratory-prepared siderite [42]. The interplanar spacing (d-spacing) of the FeCO_3_ structure was determined as 0.2796 nm [43]. The crystals have an average size of 400 nm while the mean crystallite size calculated using Scherrer’s formula was found to be about 86.6 nm for the FeCO_3_. Similarly, Nassar et al. (2016) hydrothermally synthesized FeCO_3_ structures using iron sulfate: ascorbic acid: ammonium carbonate (in a ratio of 1:3:6) at 140 °C after 3 h. They achieved FeCO_3_ structures with an average crystallite size of 80 nm [38]. Furthermore, the XRD spectrum of the FeCO_3_ catalyst was examined after its use in the EF process. As shown in Figure 2b, any distinct peaks corresponding to iron oxides or other impurities have not been observed, and the main diffraction peaks have remained intact, although of slightly reduced intensity. Combined with the SEM image observed earlier (Figure 1c,d), it can be supported that the FeCO_3_ structures remained largely intact during use.

**Figure 3:**
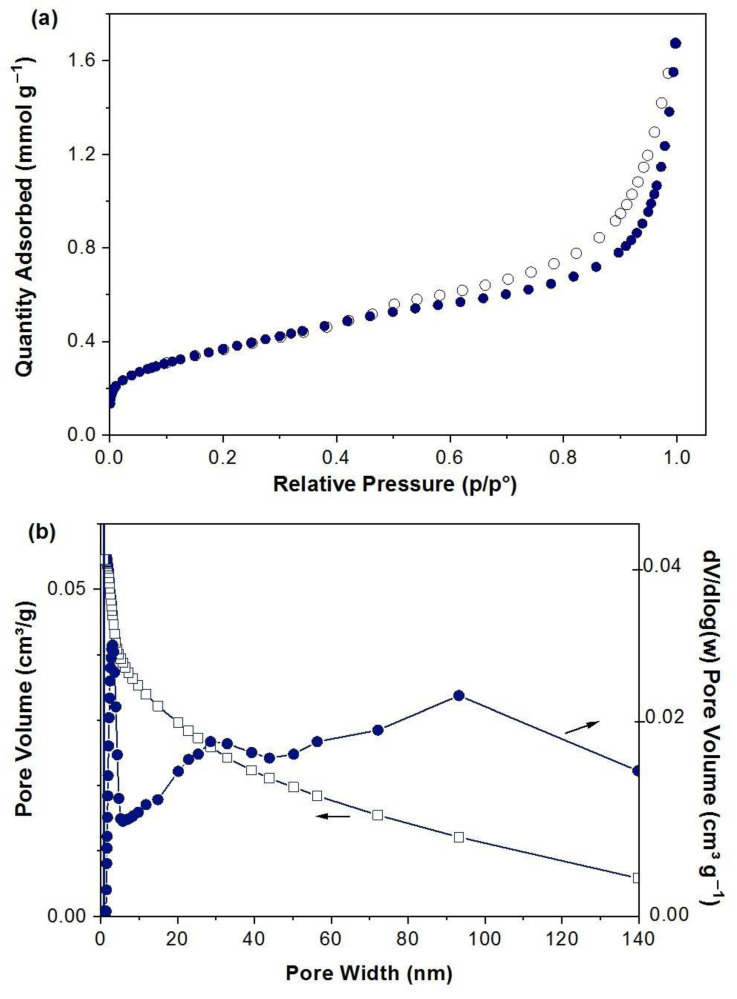
(**a**) N_2_ adsorption isotherm and (**b**) pore volume distribution against pore width for FeCO_3_.

According to the IUPAC classification, the N_2_ adsorption–desorption isotherm of FeCO_3_ resembles that of a Type IV isotherm (Figure 3a). This type of isotherm is observed in mesoporous materials and contains hysteresis (p/p^0^ = 0.5–1.0), indicating slit-like pores. The BET and Langmuir surface areas of FeCO_3_ were calculated as 29.09 and 37.07 m^2^ g^−1^, respectively. The average pore width according to Barrett–Joyner–Halenda (BJH) method and total pore volume were determined at 8.1 nm and 0.0317 cm^3^ g^−1^, respectively. Figure 3b highlights the BJH cumulative pore volume distribution against pore width for FeCO_3_.

**Figure 4:**
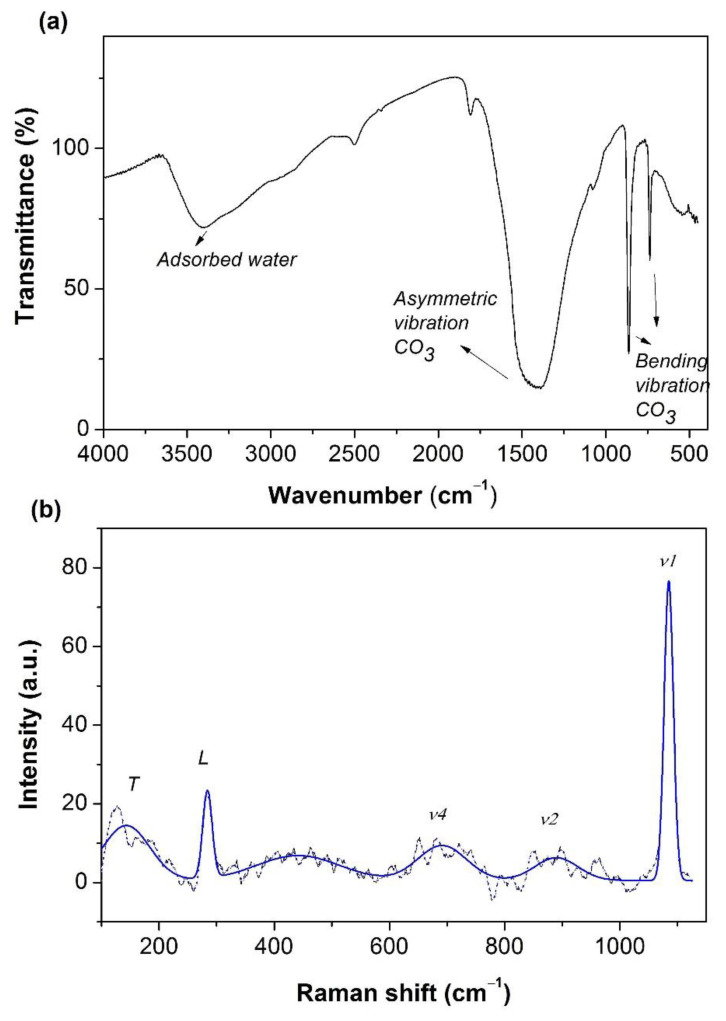
(**a**) FT-IR and (**b**) Raman spectrum of FeCO_3_.

The FT-IR spectrum of the FeCO_3_ catalyst is shown in Figure 4a. The broadband observed around 3400 cm^−1^ belongs to the O-H stretching vibrations. The broad and intense band at 1390 cm^−1^ is attributed to the asymmetric stretching vibrations of the CO_3_ group. Moreover, the bending vibrations of carbonate can be seen at 863 cm^−1^ and 738 cm ^−1^ [30,38,44]. In the Raman spectrum of the FeCO_3_ structure, the characteristic symmetrical stretching vibration (ν_1_), the out-of-plane band (ν_2_), and in-plane bending (ν_4_) peaks of the CO_3_^2−^ ion were observed at 1086 cm^−1^, 854 cm^−1^, and 720 cm^−1^, respectively (Figure 4b). Moreover, the bands belonging to the translational lattice mode (T), and librational lattice mode (L) were observed at 181 cm^−1^ and 284 cm^−1^, respectively [45,46]. Therefore, all the above analyses confirm the successful synthesis of pure FeCO_3_ crystals using the facile hydrothermal method.

### 2.2. Oxidation of p-Benzoquinone by the Electro-Fenton Process

**Figure 5:**
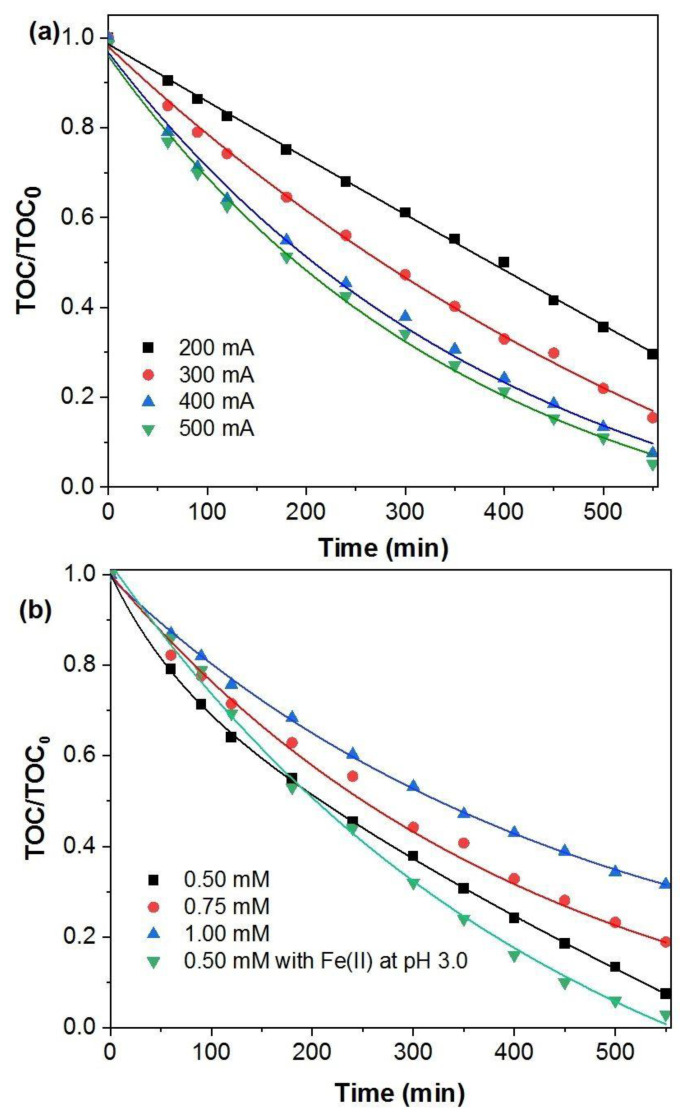
Effect of (**a**) current ([BQ]_0_ = 0.5 mM) and (**b**) initial concentration of BQ on TOC removal efficiency at 400 mA. Operating conditions: [FeCO_3_] = 0.5 g L^−1^, pH = 5.6, V = 200 mL.

The effect of the applied current on the oxidation of *p*-benzoquinone by the EF process using the FeCO_3_ catalyst is presented in Figure 5a. It can be seen that the applied current of 200 mA was inadequate due to insufficient hydroxyl radical production for the mineralization of 0.5 mM BQ solution. By raising the applied current to 300 mA, the mineralization efficiency was increased from 31 to 50% due to the enhanced ^•^OH production. At the same time, the increase in the current has improved the efficiency of the Fenton reaction by causing enhancement of the Fe^3+/^Fe^2+^ cycle [47,48]. At the 400 and 500 mA applied currents, the comparable total organic carbon (TOC) reduction values (~90%) might be reasonably explained by the increasing occurrence of side reactions. These reactions are anodic oxygen discharge (Equation (10)), cathodic hydrogen evolution (Equation (11)), and secondary reactions of ^•^OH (Equation (12)) [13]. Therefore, 400 mA can be considered the optimum applied current under our experimental setup.

2H_2_O → 4H^+^ + O_2(g)_ + 4e^−^ (10)

2H^+^ + 2e^−^ → H_2(g)_ (11)

2^•^OH → O_2(g)_ + 2H^+^ +2e^−^ (12)

The concentration of BQ in the solution is expected to affect the efficiency of the EF process, so three different initial concentrations were studied at 400 mA applied current. Figure 5b showed that increasing the BQ concentration had a negative effect on TOC reduction. At the BQ concentrations of 0.5, 0.75, and 1.0 mM, mineralization of 62%, 56%, and 47% was achieved after 300 min, respectively. At the end of the process (550 min), ~90% of 0.5 mM BQ solution was mineralized. This is probably due to the increased number of BQ and intermediate molecules that react with the hydroxyl radical, thus reducing the efficiency of the process [49,50].

The mineralization of 0.5 mM BQ solution at pH 3 in the presence of 0.35 mM (~19.55 mg L^−1^) Fe^2+^ ions by the homogeneous electro-Fenton control experiment is depicted in Figure 5b. It can be seen that the use of Fe^2+^ ions in the homogeneous control experiment achieved a near-complete mineralization (>97%) after 550 min which is comparable to the 90% mineralization achieved with FeCO_3_ heterogeneously at the same time.

**Figure 6:**
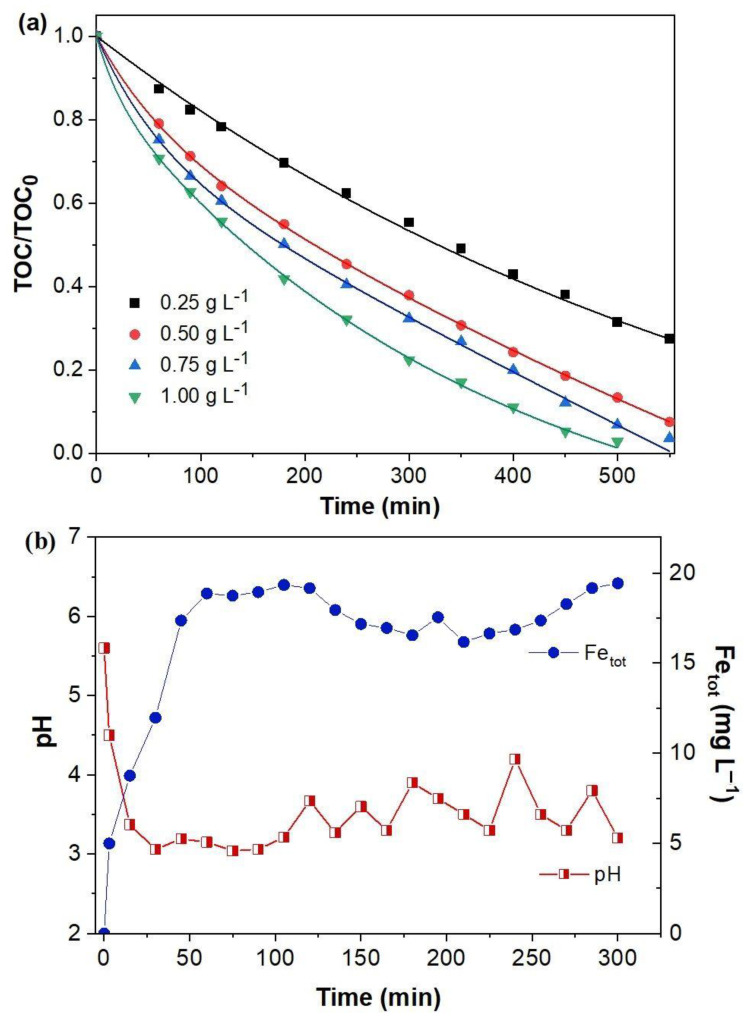
(**a**) Effect of catalyst amount at 300 mA on TOC removal efficiency and (**b**) pH change and Fe ion release during the EF process at 400 mA. Operating conditions: [BQ]_0_ = 0.5 mM, [FeCO_3_] = 0.5 g L^−1^, pH = 5.6, V = 200 mL.

From Figure 6a, BQ mineralization was seen depending on the FeCO_3_ catalyst dosage, and the increase in the mineralization efficiency due to the rise in the quantity of the catalyst was quite evident. While the TOC removal efficiency at 360 min was increased from 54 to 65% by increasing the catalyst dose from 0.25 to 0.50 g L^−1^, 84% TOC removal was achieved using 1.00 g L^−1^ of catalyst. When 1 and 0.75 g L^−1^ were used as catalyst doses, 95% mineralization was reached after 500 and 550 min, respectively, while lower yields were observed at other doses. The increase in the amount of catalyst provided an increase in the absolute number of active sites, therefore, promoting the production of more hydroxyl radicals.

The total concentration of iron ions released into the solution with the change of pH during the oxidation of BQ with 0.5 g L^−1^ FeCO_3_ catalyst is shown in Figure 6b. Until now, EF experiments were carried out at pH 5.6, which is the natural pH value of the BQ solution, without adjusting the pH value. The solution pH value decreased to 3 in the first 30 min and remained practically stable in the range of 3–4 throughout the 300 min of electrolysis. The solubility of FeCO_3_ in water is low (pK_sp_ = 10.67). However, in the reaction between FeCO_3_ and hydrogen peroxide under neutral conditions (Equation (13)), Fe^2+^ was oxidized to Fe^3+^, and the medium became acidic. At the same time, Fe(OH)_3_ and carbonic acid were formed as a result of the reaction of FeCO_3_ with oxygen (Equation (14)) [51]

FeCO_3_ + H_2_O_2_ → Fe(OH)_3_ + CO_2_+ O_2_ + H^+^ (13)

FeCO_3_ + 0.25O_2_ + 2.5H_2_O → Fe(OH)_3_ + H_2_CO_3_ (14)

As seen from the SEM images of used FeCO_3_, the rhombohedral structures were degraded, and Fe^3+^ ions were released into the solution. Compared to the 0.1–0.5 mM Fe^2+^ ions used in the homogeneous electro-Fenton control experiment, the 19.55 mg L^−1^ (0.35 mM) iron ion concentration that leached into the solution falls within this range. The efficiency of BQ mineralization carried out at pH 3 using Fe^2+^ ions at this concentration has been discussed previously and is given in Figure 5b. As seen in Figure 6b, after 1 h, an equilibrium was reached in FeCO_3_ dissolution and the total Fe^2+^ concentration remained practically constant. The Fe^3+^ ions that pass into the solution according to Equations (13) and (14) are reduced at the cathode (Equation (5)) and participate in the Fenton reaction during the EF process, contrary to the Fenton process. In addition, Fe^3+^ ions give the hydroperoxyl radical (*E*^0^ (HO_2_/H_2_O_2_) = 1.46 V/SHE), which is a weaker oxidant than the hydroxyl radical, in their reaction with hydrogen peroxide following Equation (15) [36]

Fe^3+^ + H_2_O_2_ → Fe^2+^ + HO_2_^•^ +H^+^   *k* = 0.002–0.01 M^−1^s^−1^ (15)

**Figure 7:**
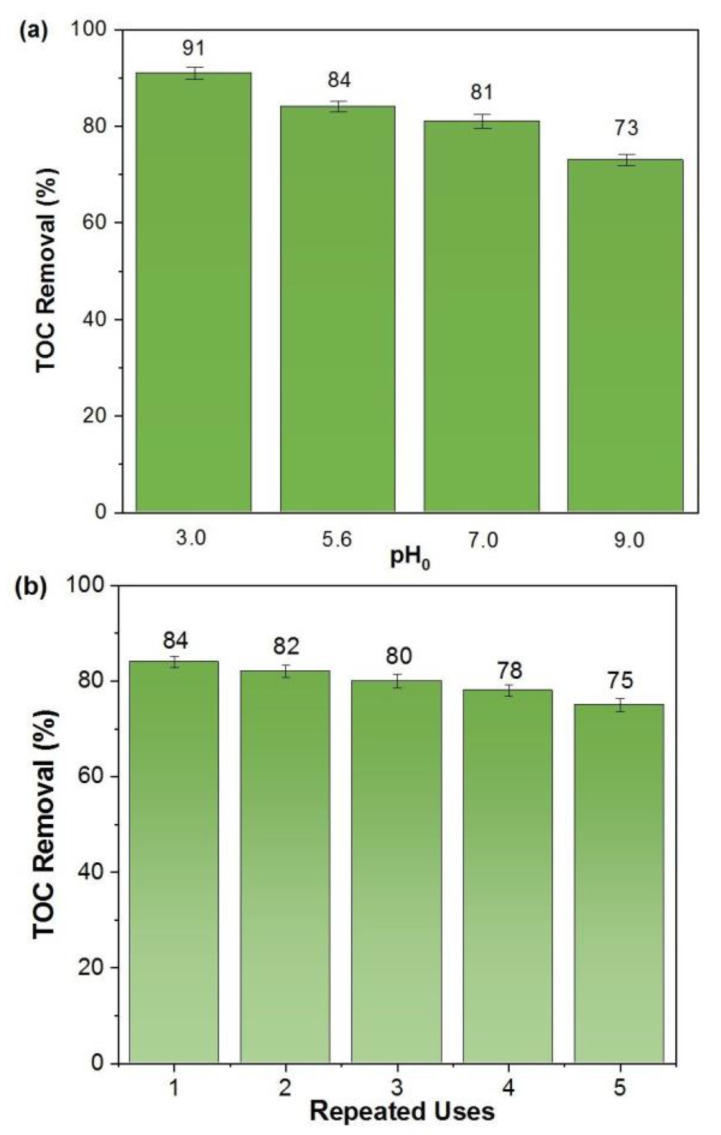
(**a**) The effect of initial pH and (**b**) recycle and reuse of FeCO_3_ (pH = 5.6). Operating conditions: [BQ]_0_ = 0.5 mM, [FeCO_3_] = 1.0 g L^−1^, I = 300 mA, t = 6 h.

The catalytic effect of FeCO_3_ on BQ oxidation by the EF process at different pH values was also examined. As seen earlier in the homogeneous EF experiment, the best mineralization efficiency was observed at pH 3 (Figure 7a). This result can be explained by Equation (16). Under acidic conditions, Fe^2+^ ions pass into the solution and participate directly in the Fenton reaction.

FeCO_3_ + 2H^+^ → Fe^2+^ + CO_2_ + H_2_O (16)

The 91% of mineralization efficiency was achieved when the pH of the BQ solution was adjusted to 3.0. TOC removal efficiencies close to each other were obtained as 84 and 81% at pH 5.6 and 7.0, respectively. When compared to pH 3.0, very effective mineralization was achieved at near-neutral pH values in the EF process with FeCO_3_. The observed decrease in TOC removal at pH 9.0 agreed with earlier results [52]. Siderite shows a very stable structure between pH 5–8 [53], but iron hydroxide precipitation realizes at high pH values on the catalyst surface and results in catalyst poisoning [54,55]. Acisli et al. (2017) investigated the effect of pH on the oxidation of Reactive Yellow 81 dye using milled natural siderite by ultrasound assisted-Fenton process and determined the most effective pH value as 3.0 [30]. As a different result, they reported that when the solution pH increased from 3.0 to 4.0, the removal percentage decreased significantly from 92 to 21%. This significant decrease in removal efficiency might be explained by the inadequacy of the Fe^3+^/Fe^2+^ cycle in the Fenton process and ^•^OH production. On the other hand, effective degradation was obtained in the pH 3–9 range using high siderite amount (6 g L^−1^) and H_2_O_2_ dosages (100 mM) for a low concentration of sodium sulfadiazine (10 mg L^−1^) in the Fenton method [52]. Consequently, while the homogeneous EF process due to free Fe ions passing into the environment at the pH value of 3.0 and 5.6 (the natural pH value of BQ solution) is effective, the heterogeneous EF process is dominant at basic pH values (Equation (17)). In case the solution pH value is 9.0, the Fe ion concentration is 2.8 mg L^−1^, supporting this statement.

Fe^II^_solid_ + H_2_O_2_ → Fe^III^_solid_ + ^•^OH + OH^−^ (17)

After using the FeCO_3_ catalyst, it was filtered, washed, and dried. Then its effectiveness in the EF process was tested in five successive runs (Figure 7b). XRD analysis of the used FeCO_3_ catalyst showed that no structure was formed to passivate the catalyst surface. A certain amount of FeCO_3_ had dissolved and passed into the aqueous medium. A significant decrease was not observed in the effectiveness of the catalyst after five uses. This catalyst, which was easy to prepare, reusable, and most importantly, can be used in near-neutral solutions without pH adjustment, has high applicability as an alternative to pyrite minerals in the homogenous/heterogeneous EF process.

Reducing the concentration of the target pollutant during the process does not mean the removal of the pollution, so TOC removal was followed. Therefore, the decrease in BQ concentration and degradation products during the process was followed by HPLC analysis. Decomposition products were determined by comparing the retention times of the standards. In the EF process performed with the 1 g L^−1^ of FeCO_3_ catalyst at pH 5.6 and 400 mA constant current, it was observed that the 0.5 mM BQ was completely degraded within 30 min. During the BQ degradation, hydroquinone and aliphatic carboxylic acids such as maleic, fumaric, formic, and acetic acids were determined as degradation products.

It should be mentioned that under real conditions, the presence of natural organic matter and chloride ions, commonly found in natural waters and wastewater, may affect the degradation of BQ. In a recent study by Cai et al. (2022), the presence of Cl^−^ dramatically enhanced the degradation of acetaminophen because Cl^−^ reacted with peroxymonosulfate to generate hypochlorous acid and chlorine radicals [56]. However, humic acid had the opposite effect acting as a radical scavenger. The above observations were confirmed by Li et al. (2022) who tested acetaminophen degradation in a heat/peroxymonosulfate system using 10–30 mM Cl^−^. At lower Cl^−^ concentrations, the inert B(OH)_3_OCl· was formed, thus reducing the formation of the chlorine radical and consequently slowing down the degradation of acetaminophen [57].

## 3. Materials and Methods

### 3.1. Materials

Reagent grade *p*-benzoquinone (C_6_H_4_O_2_) was purchased from Sigma-Aldrich and used without further purification. Anhydrous sodium sulfate (Na_2_SO_4_), urea (NH_2_CONH_2_), iron (II) sulfate heptahydrate (FeSO_4_·7H_2_O), hydroquinone (HQ), maleic acid, formic acid, fumaric acid, and acetic acid were supplied by Merck. Sodium dodecyl sulfate (SDS, NaC1_2_H_25_SO_4_ > 85%) was purchased from TCI Chemicals. The solutions were prepared with high-purity water obtained from a Millipore Milli-Q system with resistivity >18 MΩ cm.

### 3.2. Synthesis of FeCO_3_ Catalyst

FeCO_3_ catalyst was synthesized by the hydrothermal method described in detail in Zhang et al., 2015 [58]. All synthesis processes were carried out in an alloy 316 stainless steel reactor. For the synthesis of FeCO_3_, 5.6 g FeSO_4_·7H_2_O, 0.2 g SDS, and 2.4 g urea were added to 40 mL ultrapure water, respectively, and mixed for 15 min. After the reactor was closed, the air in the reactor was removed by purging with N_2_ gas. Following the adjustment of the internal pressure of the reactor with N_2_ gas, when the reactor reached the determined temperature, hydrothermal synthesis was carried out by stirring the mixture at 400 rpm for 240 min. Using the reagent quantities mentioned above at pH 3.6, temperature and pressure were applied at 140 °C and 20 bar. Then, the reactor was cooled to room temperature in a water bath, and the synthesized FeCO_3_ was filtered and washed several times with deionized water and ethanol, respectively. Finally, the product was dried at 60 °C for 24 h. The formation mechanism of FeCO_3_ involves the reactions described by the following Equations (18)–(24) [37,58]

FeSO_4_ → Fe^2+^ + SO_4_^2−^ (18)

CO(NH_2_)_2_ + H_2_O → 2NH_3_ + CO_2_ (19)

NH_3_ + H_2_O → NH_3_·H_2_O → NH_4_^+^ + OH^−^ (20)

CO_2_ + H_2_O → H_2_CO_3_ → 2H^+^ + CO_3_^2−^ (21)

Fe^2+^ + CO_3_^2−^ → FeCO_3_ (22)

Fe^2+^ + 2OH^−^ → Fe(OH)_2_ (23)

Fe(OH)_2_ + CO_3_^2−^ → FeCO_3_ + 2OH^−^ (24)

### 3.3. Characterization of FeCO_3_

The morphology and elemental composition of the FeCO_3_ catalyst were determined using a scanning electron microscope equipped with energy-dispersive X-ray spectroscopy (FEI Quanta 650, FEG-SEM). X-ray diffraction patterns were obtained with a PANalytical Empyrean brand powder device at the 2-theta angle between 10–90°. The FT-IR spectrum of FeCO_3_ was recorded in a Jasco FT-IR-6700 spectrophotometer using the KBr technique within the range of 400–4000 cm^−1^. Raman spectra were recorded with a Renishaw inVia Qontor using a 532 nm 50 mW diode laser (M2 < 1.1, beam divergence < 0.45 mrad) with 1800 line/mm grating and an x50 long working distance objective lens (Leica HCX PL FLUOTAR, WD = 8 mm, NA = 0.55). The N_2_ adsorption isotherm was measured at −196 ℃ using a Micromeritics Tristar Orion II 3020 surface area and porosimetry analyzer. Brunauer–Emmet–Teller (BET) method was used to analyze the surface area of the FeCO_3_ catalyst. The amount of Fe ions leaching into the solution was determined quantitatively by Perkin Elmer NEXION 2000 P brand inductively coupled plasma-mass spectrometer (ICP-MS).

### 3.4. Electro-Fenton Experiments and Analytical Procedures

Electro-Fenton experiments were performed in a single-compartment cell. Carbon felt cathode (15 cm × 5 cm × 1 cm, Carbone Lorraine, France) and platinum anode (20 cm^2^, Sigma-Aldrich) electrodes were placed 2 cm apart. Sodium sulfate (50 mM) was used as the supporting electrolyte, and the pH of the solution was adjusted with a pH meter (WTW 3110) using either 0.3 M H_2_SO_4_ or 0.3 M NaOH solution. The solution was saturated with O_2_ gas 15 min before the current was applied, and O_2_ was continuously bubbling and compressed to keep the concentration constant throughout electrolysis. Then the constant current was applied in the range of 100–500 mA by an MCH 303 D-II dual power supply. At the same time, a conventional homogeneous electro-Fenton experiment was carried out using 0.5 mM FeSO_4_ at pH 3 for control purposes.

The mineralization of the BQ solutions during electrolysis was determined by measuring the total organic carbon (TOC) value with a Shimadzu TOC-L_CPH_ analyzer after passing the solution through a 0.22 μm syringe filter (PTFE, hydrophilic). The BQ and HQ structures were monitored by a high-performance liquid chromatographer fitted with a C_18_ column (Lichrospher, 250 mm × 4 mm, 5 µm) and photodiode array detector (254 nm). The mobile phase consisted of acetonitrile/water mixture (60/40, *v*/*v*), and 1 mL min^−1^ flow rate was applied. For the separation of organic acids intermediates, the Grace Prevail Organic acid column (250 mm × 4.65 mm, 5 µm) was used at 210 nm with 25 mM KH_2_PO_4_ solution (adjusted pH 2.5) as mobile phase (0.5 mL min^−1^).

## 4. Conclusions

This study systemically investigated the potential catalytic effect of rhombohedral FeCO_3_ in the homogenous/heterogeneous electro-Fenton degradation of the emerging contaminant *p*-Benzoquinone. Following the successful hydrothermal synthesis of FeCO_3_ at low temperatures, its catalytic behavior was investigated in the pH range of 3–9. At the lower pH range of 3–4, FeCO_3_ released Fe^2+^ ions into the solution, thus creating homogeneous catalytic conditions for the electro-Fenton oxidation of BQ. As the pH of the solution was raised, the release of Fe^2 +^ ions was largely diminished; however, the catalytic efficiency of FeCO_3_ was comparable, achieving high mineralization rates. As a result, the reusability, pH-regulating capacity, and stability of FeCO_3_ catalyst are promising for the application of the catalyst in heterogeneous electro-Fenton degradation of recalcitrant contaminants.

## Data Availability

Not applicable.

## Sample Availability

Samples of the compounds are not available from the authors.
